# Lithuania’s experience in reducing caesarean sections among nulliparas: the impact of the quality improvement course

**DOI:** 10.1186/s12884-020-2806-5

**Published:** 2020-03-12

**Authors:** Justina Kacerauskiene, Meile Minkauskiene, Tahir Mahmood, Egle Bartuseviciene, Dalia R. Railaite, Arnoldas Bartusevicius, Mindaugas Kliucinskas, Laima Maleckiene, Jonas Ulevicius, Laura Liubiniene, Kastytis Smigelskas, Kornelija Maciuliene, Grazina Drasutiene, Diana Ramasauskaite, Ruta J. Nadisauskiene

**Affiliations:** 1 Lithuanian University of Health Sciences, Eiveniu str. 2, 50167 Kaunas, Lithuania; 2 Victoria Hospital, Kirkcaldy, Fife, Scotland KY2 5AH UK; 3 Vilnius University, Universiteto str. 3, 01513 Vilnius, Lithuania

**Keywords:** Caesarean section, Rate, Quality improvement course, Intervention, Nulliparas

## Abstract

**Background:**

To evaluate the role of the quality improvement course (QIC) to reduce the caesarean section (CS) rate among nulliparas (Robson groups 1 and 2) and to find out which group of women have reduced the CS rate following attendance at the course.

**Methods:**

The QIC was organized in 2015. For the evaluation of the CS rate after the OIC, deliveries from the selected hospitals in 2014 and 2016 were compared using MS EXCEL and SPSS 23.0.

**Results:**

Nulliparas accounted for 44.6% (3585/8046) and 42.9% (3628/8460) of all the deliveries in 2014 and 2016 years, respectively. The CS rate among nulliparas decreased from 19.0% (665/3502) in 2014 to 16.8% (593/3526) in 2016 (*p* = 0.018). The greatest decrease in absolute contribution to the overall CS rate was recorded in group 1 (*p* = 0.08). Perinatal mortality was 3.1 in 2014 and 3.9 in 2016 per 1000 deliveries (*p* = 0.569).

**Conclusion:**

The QIC has helped to reduce the CS rate among nulliparas without a negative influence on perinatal mortality. The greatest decrease in the overall CS rate was recorded among nulliparous women who were treated with oxytocin and managed to reach a full cervical dilatation.

## Background

The caesarean section (CS) rate in Lithuania increased from 9.49% in 1995 to 26.01% in 2012 [1] and a similar trend has been reported worldwide [2]. Nevertheless, the medical justification for a CS is not always clear and sometimes it is even doubtful. The World Health Organization (WHO) has proposed non-clinical interventions to reduce the CS rate. They are targeted at women, health-care professionals and health organizations, facilities or systems [3]. One of the strategies to reduce the CS rate is by organizing “Quality improvement courses” dedicated to improve clinical skills in obstetrics. Chaillet and Dumont reported in their meta-analysis the impact of various interventions on the CS rate. There were four studies dedicated to analyze the effect of quality improvement strategies. All of them have reported a positive impact on the reduction of the CS rate [4]. Despite the fact that these strategies address all delivering women, but special attention should be paid to first-time mothers. Nulliparous women have a greater risk of adverse outcomes during their deliveries as compared with multiparous women. On the other hand, the outcome of their deliveries is of great importance because of the influence on subsequent deliveries. Many studies have shown that nulliparous women with a single cephalic, term pregnancy in spontaneous or induced labour or those delivered by an elective CS (nulliparas) and women with previous CSs, make the greatest contribution to the overall CS rate [5–14]. Therefore, a CS performed for a nulliparous woman can influence the CS rate in subsequent pregnancies. The first time it increases the CS rate among nulliparas and then it increases the risk of a repeat CS from 3% to 50–60% during second pregnancy [8] when compared with multiparous women without previous CSs. Therefore, a reduction in the number of CSs among nulliparas will ultimately reduce the number of CSs among multiparas in the future.

A national quality improvement course (QIC) for obstetrical skills improvement was organized for obstetric staff involved in intra-partum care in 2015 in Lithuania. It was part of the Lithuanian–Swiss Cooperation Programme [15]. The aim of this study was to evaluate the role of the QIC as a national intervention in reducing the CS rate among nulliparous women. The objective of this study was to identify the group of women where the CS rate was reduced following attendance at the QIC.

For a unified classification of delivering women, the Robson classification (also known as the 10-group classification system) was proposed by the WHO (Table 1) [16, 17]. It is based on a few of obstetric variables (parity, previous CS, onset of labour, number of fetuses, gestational age and fetal lie and presentation) and might be applied to every woman who gives birth [17]. Therefore, based on this classification we have included in this study all nulliparous women with a single cephalic, term pregnancy in spontaneous (group 1) or induced labour (group 2A) or an elective CS (group 2B). Nulliparous women falling within groups 2A or 2B formed group 2. Women from groups 1 and 2 were treated as nulliparous in this study.

**Table 1:** The 10-group classification system

No.	Group
Group 1	Nulliparous, single cephalic, 37 weeks, in spontaneous labour
Group 2A	Nulliparous, single cephalic, 37 weeks, induced labour
Group 2B	Nulliparous, single cephalic, 37 weeks, CS before labour
Group 3	Multiparous (excluding prev. CS), single cephalic, 37 weeks, in spontaneous labour
Group 4A	Multiparous (excluding prev. CS), single cephalic, 37 weeks, induced labour
Group 4B	Multiparous (excluding prev. CS), single cephalic, 37 weeks, CS before a labour
Group 5A	Previous CS, single cephalic, 37 weeks, induced labour
Group 5B	Previous CS, single cephalic, 37 weeks, CS before labour
Group 5C	Previous CS, single cephalic, 37 weeks, in spontaneous labour
Group 6	All nulliparous breeches
Group 7	All multiparous breeches (including prev. CS)
Group 8	All multiple pregnancies (including prev. CS)
Group 9	All abnormal lies (including prev. CS)
Group 10	All single cephalic, ≤ 36 weeks (including prev. CS)

### Objectives

To evaluate the role of the quality improvement course (QIC) to reduce the caesarean section (CS) rate among nulliparas (Robson groups 1 and 2) and to find out which group of women have reduced the CS rate following attendance at the course.

## Methods

A multifaceted intervention was performed in 2012–2016 in Lithuania (as stated in the description in our previous paper [18]). This paper presents the change in the CS rate after the national educational course.

The national QIC was held in Lithuania in 2015. No similar national courses had been organized previously. The QIC took place in different cities and was organized several times. The course was dedicated to delivery-related staff from all Lithuanian hospitals providing obstetrical care. All these hospitals had their representatives attending the course. They included not only obstetricians and gynaecologists but also midwives, neonatologists and nurses. The aim of the QIC was to ensure that all Lithuanian hospitals providing obstetrical care had the same obstetrical knowledge and provided standardized care in managing the same obstetrical situations. The course consisted of educational sessions and practice drills. The core of educational sessions was a file of evidence-based obstetrical guidelines that were distributed nationally [19] and are openly accessible all the time. After updating evidence-based guidelines, the participants practiced their obstetrical skills including vacuum extraction, shoulder dystocia, evaluation of cardiotocogram, and teamwork etc. In order to finish the course, all the participants had to pass a knowledge and skills examination.

For the evaluation of the CS rate among nulliparas after the national intervention, the deliveries of nulliparous women in the selected hospitals in 2014 and 2016 were compared. The principles hospital selection and calculation of the sample size are described in the article by Kacerauskiene et al. [18]. There were three level IIA hospitals (with 1604 deliveries in total in 2014 and 1632 in 2016), one level IIB hospital (with 3075 deliveries in 2014 and 3197 in 2016) and one level III hospital (with 3367 deliveries in 2014 and 3631 in 2016) included in the study.

Socio-demographic, personal and specific delivery-related data were gathered using the hospitals’ delivery records, the national database of the Lithuanian Hygiene Institute and case notes. Some case notes were absent, other which did not contain the required information or were illegible, were excluded from a specific CS’s analysis. Therefore, the number of participants that represents socio-demographic and personal data might be different to the number that represents delivery-related data.

### Outcomes

As stated in our previous paper [18], the overall CS rate was the primary outcome. Secondary outcomes included specific delivery-related data (indications for a CS, delivery outcomes, cervical characteristics at the start of delivery stimulation or the decision to perform a CS) and newborn-related outcomes (birth weight and Apgar scores). Perinatal mortality was calculated too. Indications for a CS were not re-checked and information was obtained by medical chart review. Socio-demographic and personal data (marital status, living area, education, age, maternal diseases (all diseases that are not related to pregnancy, i.e. eye, ear, pulmonary, cardiovascular, renal and other pathologies)) as well as pregnancy-related conditions (i.e. gestational hypertensive disorders, gestational cholestasis, gestational diabetes etc.) were assessed as potential risk factors and were included in the statistical analysis too.

Robson et al. have proposed a new classification of indications for a CS in nulliparas assigned to group 1 [10]. We have developed ***a new modified classification***. The main difference was that various conditions (i.e. umbilical cord prolapse after the spontaneous onset of labour, placental abruption etc.) were grouped within a separate group. For the vast majority of such deliveries CS is unavoidable. Therefore, no specific strategies can be created to manage such a delivery vaginally. The other reason for modifying the classification was different management of delivery itself: a different perception of latent and active phases, oxytocine dosage etc. A new modified classification included whether variables such as augmentation with oxytocin, dilatation of the cervix (complete cervical dilatation (CCD) and incomplete cervical dilatation (< 10 cm) (ICD)), suspected fetal compromise (FC) and other conditions such as placental abruptions, umbilical cord prolapse, and elective CSs with spontaneous labour. The indications for CS in group 1 were classified as dystocia, FC and others.

### Participants

All nulliparous women who gave birth in the course of the study in the selected hospitals and were ascribed to groups 1 or 2 according to the Robson classification.

### Statistical analysis

Programs *MS Excel* and *IBM SPSS Statistics 23.0 for Windows* were used to analyze the data. Health care institutions providing obstetrical care of different levels were represented by 3 groups of hospitals: level IIA, level IIB and level III. There were 28,230 deliveries at the beginning of the multifaceted intervention in 2012 in Lithuania. In order to detect a 5% change in the CS rate with a power of 80% and a significance level of 5%, a minimum of 1197 deliveries had to be included in each group providing obstetrical services at a different level. From these deliveries only nulliparous women falling within groups 1 and 2 according to the TGCS were selected and the outcomes of their deliveries in 2014 and 2016 were compared. The chi-squared test was conducted to compare the study groups. In those cases where the assumptions for the chi-squared test were not met, the Fisher’s exact test was applied.

## Results

There were 8046 and 8460 deliveries in 2014 and 2016, respectively. Nulliparas accounted for 44.6% (3585/8046) of all deliveries in 2014 and 42.9% (3628/8460) in 2016. Sociodemographic and personal data are presented in Table 2.

**Table 2:** Socio-demographic and personal data

	2014	2016	*p* value
**Living area**			
urban, n (%)	2886 (80.5)	2967 (81.8)	*p* = 0.165
rural, n (%)	699 (19.5)	661 (18.2)	
**Education**			
basic, primary and secondary, n (%)	1649 (46.0)	1600 (44.1)	*p* = 0.106
higher, n (%)	1936 (54.0)	2028 (55.9)	
**Marital status**			
single, n (%)	1089 (30.4)	1049 (28.9)	*p* = 0.174
married, n (%)	2496 (69.6)	2579 (71.1)	
**Age**			
< 20, n (%)	1142 (31.9)	1016 (28.0)	*p* = 0.002
20–34, n (%)	2224 (62.0)	2377 (65.5)	
≥ 35, n (%)	219 (6.1)	235 (6.5)	
**Maternal diseases,** n (%)	124 (3.5)	122 (3.4)	*p* = 0.826

Overall, 97.7% (3502/3585) of all case histories were analyzed in 2014. In 2016, this number was 97.2% (3526/3628). The CS rate among nulliparas decreased from 19.0% (665/3502) in 2014 to 16.8% (593/3526) in 2016 (*p* = 0.018) (Table 3). The greatest decrease in absolute contribution to the overall CS rate was recorded in group 1 (*p* = 0.08). It decreased from 9.7% (340/3502) in 2014 to 8.5% (300/3526) in 2016. The number of instrumental deliveries decreased too: from 2.9% (102/3502) in 2014 to 2.6% (90/3526) in 2016 (*p* = 0.352), although the change was non- significant.

**Table 3:** The CS rate in 2014 and 2016

	Number of CSs in the total number of women in each group		Relative group size (%)		CS rate in each group (%)		*p* value	Absolute contribution to the overall CS rate (%)		*p* value
	2014	2016	2014	2016	2014	2016		2014	2016	
Group 1	340/2698	300/2590	77.0 2698/3502	73.5 2590/3526	12.6	11.6	*p* = 0.258	9.7340/3502	8.5300/3526	*p* = 0.08
Group 2	326/804	293/936	23.0804/3502	26.5936/3526	40.5	31.3	*p* < 0.001	9.3326/3502	8.3293/3526	*p* = 0.139
Group 2A	220/698	211/852	19.9698/3502	24.2852/3526	31.5	24.8	*p* = 0.003	6.3220/3502	6211/3526	*p* = 0.603
Group 2B	106/106	82/82	3.0106/3502	2.3 82/3526	100	100	–	3.0106/3502	2.3 82/3526	*p* = 0.069
Group 1 and 2	665/3502	593/3526			19.0	16.8	*p* = 0.018	665/3502	593/3526	*p* = 0.018

The CS rate in group 1 decreased from 12.6% (340/2698) in 2014 to 11.6% (300/2590) in 2016 (*p* = 0.258). The greatest contribution to the overall CS rate in this group was made by dystocia (Table 4). The greatest reduction in this group was among women who were treated with oxytocin and managed to reach a full cervical dilatation (“CCD with oxytocin”). The peculiarities related to dystocia are presented in Table 5. The number of operations due to other indications did not change statistically significantly (Table 4).

**Table 4:** Indications for CS among women in group 1

Indication	Absolute contribution to the CS rate in group 1, %, 2014	Absolute contribution to the CS rate in group 1, %, 2016	*p* value	Absolute contribution to the overall CS rate, %, 2014	Absolute contribution to the overall CS rate, %, 2016	*p* value
**FC** (without oxytocin)	15.9 54/340	17.3 53/294	*p* = 0.472	1.5 54/3502	1.4 53/3526	*p* = 0.101
**Dystocia**	74.1252/340	75.5225/294	*p* = 0.484	7.2252/3502	6.3225/3526	*p* = 0.174
CCD without oxytocin	10.0 34/340	10.5 31/294	*p* = 0.818	1.0 34/3502	0.9 31/3526	*p* = 0.689
CCD with oxytocin	20.0 68/340	16.0 47/294	*p* = 0.19	2.9 68/3502	1.3 47/3526	*p* = 0.044
ICD without oxytocin	6.2 21/340	7.1 21/294	*p* = 0.624	0.6 21/3502	0.6 21/3526	*p* = 0.984
ICD with oxytocin	27.9 95/340	33.0100/294	*p* = 0.099	2.7 95/3502	2.8100/3526	*p* = 0.757
FC with oxytocin^a^	10.0 34/340	8.8 26/294	*p* = 0.617	1.0 34/3502	0.7 26/3526	*p* = 0.289
**Others**	10.0 34/340	7.1 22/294	*p* = 0.267	1.0 34/3502	0.6 22/3526	*p* = 0.101
**Total**				9.7340/3502	8.5300/3526	*p* = 0.08

^a^“FC with oxytocin” was attributed to “dystocia” because the reason why oxytocin was administered was dystocia

**Table 5:** Peculiarities of deliveries when the indication for CS is “dystocia”

	2014	2016	*p* value
**Dystocia: CCD with oxytocin**			
Cervical dilatation at the moment of oxytocin administration, cm (mean, SD)	5.7 (2.21)	5.07 (2.03)	*p* = 0.127
Waiting time passed before making the decision to perform a CS because of dystocia, min (mean, SD)	87.8 (38.7)	107.62 (73.21)	*p* = 0.095
**Dystocia: ICD with oxytocin**			
Cervical dilatation at the moment of oxytocin administration, cm (mean, SD)	4.1 (2.24)	4.38 (2.47)	*p* = 0.411
Cervical dilatation at the moment of the decision to perform a CS because of dystocia, cm (mean, SD)	5.38 (2.61)	6.16 (2.58)	*p* = 0.04
Waiting time passed before the decision to perform CS because of dystocia, min (mean, SD)	217.21 (164.47)	204.82 (162.01)	*p* = 0.06

The CS rate in group 2 decreased from 40.5% (326/804) in 2014 to 31.3% (293/936) in 2016 (*p* < 0.001). Nevertheless, the absolute contribution to the overall CS rate of this group did not change statistically significantly (*p* = 0.139) (Table 3). Although statistically insignificant, the number of elective CSs decreased from 3.0% (106/3502) in 2014 to 2.3% (82/3526) in 2016 (*p* = 0.069). This decrease was mostly attributed to the reduced number of operations because of “post-term pregnancy” (*p* = 0.016) (Table 6).

**Table 6:** Indications for operations in women with elective CSs

Indication	Absolute contribution to the CS rate in group 2B, %, 2014	Absolute contribution to the CS rate in group 2B, %, 2016	p value	Absolute contribution to the overall CS rate, %, 2014	Absolute contribution to the overall CS rate, %, 2016	p value
Placental and umbilical cord pathology	10.4 11/106	4.9 4/82	*p* = 0.168	0.3 11/3502	0.1 4/3526	*p* = 0.069
Fetus related conditions	11.3 12/106	15.9 13/82	*p* = 0.363	0.3 12/3502	04 13/3526	*p* = 0.857
Maternal diseases	49.0 52/106	61.0 50/82	*p* = 0.103	1.5 52/3502	1.4 50/3526	*p* = 0.667
Suspected fetal macrosomia	9.4 10/106	4.9 4/82	*p* = 0.238	0.3 10/3502	0.1 4/3526	*p* = 0.105
Preeclampsia	4.7 5/106	7.3 6/82	*p* = 0.453	0.1 5/3502	0.2 6/3526	*p* = 0.772
Post-term pregnancy	15.1 16/106	6.1 5/82	*p* = 0.052	0.5 16/3502	0.1 5/3526	*p* = 0.016
Total				3.0106/3502	2.3 82/3526	*p* = 0.069

No change in perinatal mortality was detected (*p* = 0.569). In 2014, perinatal mortality was 3.1 and in 2016 it was 3.9 in 1000 deliveries (Table 7). A statistically significant difference was detected in the Apgar score after 5 min in 2016 (*p* < 0.001). It might be attributed to a high number of newborns and might be of clinical significance (Table 7).

**Table 7:** Newborn related outcomes

Indicator	2014	2016	*p* value
**Newborn weight**			
< 3000 g, n (%)	429 (12.0)	371 (10.2)	*p* = 0.01
3000–3999 g, n (%)	2691 (75.1)	2721 (75.0)	
≥ 4000 g, n (%)	465 (13.0)	536 (14.8)	
**Apgar score after 5 min**			
≤ 7, n (%)	6 (0.2)	26 (0.7)	*p* < 0.001
≥ 8, n (%)	3579 (99.8)	3602 (99.3)	
**Perinatal mortality**			
antepartum deaths, n	7	8	*p* = 0.478
intrapartum deaths, n	0	2	
early neonatal deaths, n	4	4	
perinatal mortality	3.1/1000	3.9/1000	*p* = 0.569

## Discussion

This national intervention, which involved implementation of obstetrical guidelines on labour care and standardized practice of obstetrical skills, which was implemented to avoid the overuse of medically unnecessary operations, helped to reduce the CS rate by 2.2%. Similar results were reported by other authors. Chaillet et al. reported a reduction by 0.7% of the CS rate after the implementation of a multifaceted intervention [6]. Beside interventions like audit and feedback, the obstetrical guidelines were a part of that intervention. Yet, the authors did not specify how each individual intervention impacted the CS rate [6]. A study from Sweden [7] has shown a positive impact of a multifaceted intervention on the reduction of the CS rate which decreased from 10% in 2006 to 3% in 2015 among nulliparas in group 1 (according to Robson). Among other interventions, obstetrical skills training, teamwork and improvement of fetal monitoring skills were similar to those in our study [7]. Wilson – Leedy et al. analyzed the impact of delivery management guidelines on the CS rate among induced or augmented labour among nulliparas and reported decreased rate from 35.5 to 24.5% after the intervention [20].

The aim of the EC in Lithuania was to improve specific theoretical and practical skills, especially those that do not occur in everyday clinical practice (i.e. instrumental deliveries and shoulder dystocia). However, the number of instrumental deliveries decreased after the course, which contradicts one of the aims of the course. A similar outcome was proposed by Sorensen et al. [21]. However, Skinner et al. found that education programs might have a positive impact on a certain skill [22].

The greatest decrease in the CS rate among nulliparas with spontaneous labour was found among women who were treated with oxytocin and managed to reach full cervical dilatation. Before the intervention, this group was the main contributor to the overall CS rate. The main reason for CSs in this group might be insufficient waiting for the delivery of the fetus. In 2014, it was 87 min. During the dystocia dedicated course session, the attendees were encouraged to prolong the waiting time during the second stage of labour. In 2016, the waiting time before the delivery of the newborn increased by 20 min, from 87 to 107 min. This could be attributed to the decreased number of CSs.

After the intervention, the greatest contribution to the overall CS rate was attributed to women who were treated with oxytocin but did not reach the full cervical dilatation. The high CS rate in this group could be explained by a couple of reasons. There is a low dose oxytocin protocol in Lithuania. It is possible that despite the administration of oxytocin, the dose was insufficient to achieve adequately strong uterine contractions to lead to full cervical dilatation in certain cases. It is equally possible that a higher titration of oxytocin dose would lead to an increase in the number of women whose fetuses could not have tolerated oxytocin stimulation. This assumption is supported by the fact that the number of women in the “FC with oxytocin” group was low; 3.5 times lower than in a hospital with a high dose oxytocin protocol [23]. Another reason might be that oxytocin is administered too early and the cervix is not yet primed enough to adequately respond to oxytocin. In our study, the mean dilatation of the cervix when oxytocin was administered in the “ICD with oxytocin” group was approximately 4 cm, while in the“CCD with oxytocin” group it was 5 cm. Therefore, it could be hypothesized that the later oxytocin is administered, the better is the chance to reach complete cervical dilatation. According to Wilson – Leedy et al., who investigated delivery outcomes for induced or augmented nulliparas, no decision to perform a CS should be made before cervical dilatation reaches 6 cm [18, 24]. This could help to reduce the CS rate. Cervical dilatation at the moment of the decision to perform CS because of dystocia increased in our study from 5.38 cm in 2014 to 6.16 cm in 2016. In spite of that, the contribution to the CS rate in ICD group did not change. One more factor ensuring a more accurate dosage of oxytocin is that during the course all the attendees were taught to use infusion pumps, which was not common before the QIC.

The CS rate in group 2 decreased statistically significantly in 2016. Moreover, the CS rate among women with induced delivery reached the lowest recommended value [8]. Despite this fact, the absolute contribution to the overall CS rate of group 2 did not change. This might be attributed to the fact that the number of women in group 2 increased and mostly by women with induced delivery. This shows that cutting the number of induced deliveries among nulliparas could have a positive impact on reduction of the CS rate.

### Strengths and limitations

The strengths of this study are the following: the organized course were available to the attendees from all Lithuanian hospitals; the obstetrical guidelines that were presented during the course are available on-line all the time and are implementing in Lithuania’s hospitals; all the data were checked and gathered by the same single study investigator, (JK), what ensured an equal classification of the deliveries; this study introduces a new classification of intrapartum caesarean sections. This enables us to analyze and compare not only the number of CSs but also the reasons for doing them.

The limitations of the study are the following: not all case notes were available for the analysis; some of the delivery-related staff from Lithuanian hospitals was not able to attend the course and no maternal outcomes are analyzed and discussed.

## Conclusions

The national quality improvement course has helped to reduce the CS rate among nulliparous without a negative influence on perinatal mortality. The greatest reduction in the CS rate was recorded for women who were treated with oxytocin and managed to reach a full cervical dilatation.

## Data Availability

The datasets used and/or analysed during the current study are available from the corresponding author on reasonable request.

## Abbreviations

CCD: Complete cervical dilatation
CS: Caesarean section
FC: Fetal compromise
ICD: Incomplete cervical dilatation
QIC: Quality improvement course
